# Farm and Animal Factors Associated with Morbidity, Mortality, and Growth of Pre-Weaned Heifer Dairy Calves in Southern Brazil

**DOI:** 10.3390/ani14223327

**Published:** 2024-11-19

**Authors:** Gabriela Olmos Antillón, Vilmar Fruscalso, Maria José Hötzel

**Affiliations:** 1 Veterinary Epidemiology Unit, Department of Clinical Sciences, Swedish University of Agricultural Sciences, Box 7054, 75007 Uppsala, Sweden; 2 Technical Department, Riograndense Association of Enterprises for Technical Assistance and Rural Extension (EMATER/RS), Porto Alegre 90150-052, RS, Brazil; 3 Laboratório de Etologia Aplicada e Bem-Estar Animal, Departamento de Zootecnia e Desenvolvimento Rural, Universidade Federal de Santa Catarina, Florianópolis 88040-900, SC, Brazil

**Keywords:** animal welfare, calf, perinatal, postnatal, smallholder

## Abstract

This study investigates morbidity, mortality, and weight gain in pre-weaned female dairy calves, which pose economic and animal welfare challenges for farms, particularly in family-run operations in southern Brazil. We aimed to identify the rates and factors associated with these outcomes in Alto Uruguai, Rio Grande do Sul, Brazil, tracking 547 calves from 70 farms across 27 municipalities from July 2015 to September 2016. We assessed calf-rearing practices, nutrition, health, and the environment using farmer questionnaires, direct observations, and systematic weight and health monitoring of female calves from birth to weaning. The association between predictors and perinatal mortality (stillbirths and deaths within 24 h), postnatal mortality (deaths from 25 h after birth to weaning), calf morbidity, and weight gain was analysed through regression models. The participating farms had 25 (9–70) (median and range) lactating cows and produced 411 (96–1631) L/d of milk (median and range). Total mortality rate was 6.8% (median 0, range 0–50%/range), of which 2.4% (median 0, range 0–50%/range) was perinatal and 4.5% (median 0, range 0–40%/range) postnatal. Average morbidity was one case for every five calves born alive (106/538), 83% of which were reported as diarrhoea cases. Weight gain was 570 ± 212 g/d (mean and standard deviation). The postnatal mortality rate was highest in the first few weeks of life and among calves with at least one case of diarrhoea. Younger and winter/spring-born calves became sick more frequently. The calves of purebred bulls, fed with cow’s milk, which received at least 4 L of milk and 0.5 kg of feed daily, had greater weight gain. The calf mortality rate was at the lower end of the range reported in international research. The main disease affecting calves was diarrhoea, and weight gain was insufficient for calves to double their weight by the time they were weaned. The results of the present study suggest that the quality of the management adopted in calf rearing in many of the family-run dairy farms of southern Brazil may be compromising the welfare, health, performance, and survival of the calves and possibly the productivity of dairy herds.

## Simple Summary

We investigated the health and growth of dairy calves in southern Brazil. Over the course of a year, we tracked 547 calves from 70 farms across 27 municipalities, focusing on factors such as farming practices, nutrition, and health care. The average pre-weaning mortality rate was 6.8%. The main disease affecting calves was diarrhoea; additionally, weight gain (570 ± 212 g/d) was insufficient for calves to double their weight by the time they were weaned. Despite differences in farm size and management, the calf morbidity and mortality rates were similar to international studies. Our findings suggest that colostrum and milk feeding, as well as the quality of the rearing environment are major challenges to be overcome in the studied region and may help explain the relatively poor productive performance and economic viability of dairy activity in many farms in the region. They can be useful for future comparisons between farms, as well as to support extensionists, consultants, and dairy farmers and to guide official programs.

## 1. Introduction

Most deaths of dairy cattle under one year of age occur in the first months of life, which makes this phase highly critical to calf health, development and future performance. International reports indicate that the mortality rate of dairy calves’ range between 2 and 10% in the perinatal period (≤24 h) and between 5 and 11% in the postnatal period (i.e., 25 h after birth until weaning) [1,2,3]. Neonatal diarrhoea, respiratory diseases, and navel infections are the most prevalent and costly diseases affecting calves [3]. It has been proposed that calves should double their weight from birth until weaning [4,5]. Poor calf development may occur due to deficient management, inadequate environment, disease, and malnutrition [6]. Reduced weight gain has been associated with failures in passive immunity transfer [6], low milk intake [7,8,9], digestive, respiratory and blood infections [10], and umbilical infections [11]. These early-life challenges affect future calf performance and significantly affect animal welfare. High rates of morbidity and mortality highlight deficiencies in management and health, contributing to calf suffering. Improving disease prevention, nutrition, and passive immunity transfer is crucial for enhancing calf welfare and better production outcomes. Ensuring proper early-life care is key to reducing mortality and morbidity, ultimately promoting healthier and more humane conditions for calves.

The milk and dairy production chain is a sector of great economic and social importance for Brazil. Milk production takes place in 98% of Brazilian municipalities, primarily on small and medium-sized farms, and it provides employment for nearly 4 million people. The country is home to more than 1 million milk-producing rural establishments [12]. Despite the importance for the issue for the dairy industry, there is a scarcity of field studies on the prevalence and associated factors to pre-weaning morbidity and mortality of female dairy calves in Brazil. Yet, regional and national data on calf mortality are crucial for identifying local challenges, tailoring management practices to specific environmental and economic conditions, and benchmarking performance within the dairy industry. The few available studies point at diarrhoea and respiratory diseases as the leading causes of death in dairy calves [13,14,15]. This observational study aimed to identify the prevalence and factors associated with mortality, morbidity and weight gain of dairy calves between birth and weaning in smallholdings typical of the country’s southern region. This information will aid in developing targeted interventions and informing on calf health and welfare on a broader scale.

## 2. Materials and Methods

This study was designed to be a prospective cohort study aimed at identifying risk factors associated with female calf morbidity and mortality. This study was carried out between July 2015 and September 2016 in the region of Alto Uruguai Gaucho (AUG) in the state of Rio Grande do Sul, Brazil, and was part of a larger project that focused on the socio-environmental characterisation of dairy calf rearing systems in pasture-based dairy farms in Brazil [16]. Rio Grande do Sul (RS) is the third largest dairy-producing state in the country, with 4.1 billion litres of milk per year in 2022. The average productivity in the state in 2021 was estimated at 4129 litres/cow/year, and milk production is present in some form on a total of 137,449 rural properties, distributed across 493 out of the 497 municipalities in the state. Among milk producers, 40,182 engage in dairy farming as a formal economic activity, with 39,991 producers directly selling raw milk to industries, cooperatives, or cheese factories [17]. According to the latest national census available, it is estimated that up to 85% of the milk produced in the state is produced in small family run pasture-based dairy units [18].

All procedures were approved by the Ethics Committees on Research on Humans (Protocol 1.344.025, 2015) and Animals (Protocol PP00962, 2015) of the Federal University of Santa Catarina. The second author, with the aid of technicians from EMATER (Associação Riograndense de Empreendimentos de Assistência Técnica e Extensão Rural do Rio Grande do Sul), collected all the data. EMATER, the RS official extension agency, provides free technical assistance for agricultural development. The services are available to any dairy producer in any state municipality.

### 2.1. Selection of Participants

This study design was based on an estimated prospective survey [19] of n = 136 dairy farmers. The estimation of farms needed for the study was performed as follows:

(1) $$n=\frac{N\cdot Z^{2}\cdot p\cdot \left(1-p\right)}{\begin{array}{c} \left(N-1\right)\cdot e^{2}+Z^{2}\cdot p\cdot \left(1-p\right) \end{array}}$$

n = sample size (i.e., number of farms);
N = population size (total number of farms in the AUG = 6759) [20];
Z = confidence (95%);
e = error (5%);
p = expected mortality (10%) [2].

The aims were to (A) attain the minimum number of farms to be surveyed to estimate dairy calf mortality in the northern RS and (B) to assess the potential factors that increase mortality at the farm level. The rule applied was to have a proportion of 10 farms per variable to be evaluated in a multivariable model. This rule is common when knowledge is scarce about the factors to be evaluated that would allow a better calculation.

Any farm in the AUG region that raised their heifer calves on the farm and attended the municipal offices of EMATER during the research period was eligible to participate. The farmers were indicated with the aid of technicians from the EMATER/RS offices most connected to the dairy activity. Initially, a total of 135 farms, located in the 32 municipalities of AUG, were part of the study.

### 2.2. Data Collection

The first author (VF), the producers, and 32 technicians from the EMATER municipal offices participated in the data collection, where each technician was responsible for the farms within their working municipality. Four farms per municipality were included in the study, with the exception of two municipalities with six farms each and one municipality with seven participating farms. Farms were selected using convenience sampling to ensure reliable participation and data collection throughout the study. At the beginning of the project, all technicians received a general orientation of the procedures, including how to train and assist farmers in the identification of disease symptomatology, performing biometrics, and reporting these findings on the forms designed for the study.

Each participating farm was contacted, and an initial visit was agreed upon. On this initial visit, farmers were fully debriefed of the purposes, needs, and risks of the project and, upon full agreement, asked to sign a free and informed consent form. Once the agreement was signed, the weighing procedure was shown to the farmer by VF and the technician involved. The procedure was then repeated by the farmer to correct for any discrepancies in the procedure. Moreover, the farmers were also trained on the identification of diseases’ symptoms and filling in the records. Thereafter, the municipal technician together with VF were in charge of accompanying the farmers in subsequent monthly visits or as necessary.

Each farm was visited 12 times (approximately once every month). Regardless of the schedule of visits, when the first calf was born, VF and the assigned local technician went to the farm to support the data collection process; thereafter, all procedures were performed mainly by the farmer. During each visit, the technicians checked the calf files, inspected the animals and their environments, and answered any queries from the farmers that arose during that month. On the first visit, VF made a socio-environmental diagnosis of the farm with special focus on the calf rearing system (nutrition, health, and environment) using a questionnaire containing multiple choice and semi-open questions. The environmental inspection was performed using a checklist. These included the features of the place of calving (shade, drainage, and hygiene) and the quality (hygiene, humidity, heat stroke, and ventilation) and type (area, bed, floor, and roof) of calf housing. Hygiene, humidity, insulation, and ventilation were classified as good, average, or poor, according to the criteria described in Table 1. All data collection was supported by the following documents (Supplementary Materials): Heifer Sheet; clinical score for bovine respiratory disease; and score for the diagnosis of sepsis. On the heifer form, the participant’s data, heifer sire’ breed, dam’s parity, birth conditions (normal birth/no assistance, birth with the assistance of 1 person, birth with the assistance of ≥2 people, or birth with via mechanical/surgical intervention), form of colostrum supplementation, liquid diet, and housing system were recorded. The dates of birth, death, and weaning; the diseases observed; and the weight at birth and weaning were also recorded. On the back of the form, any health events were recorded, with the date, name, and symptoms of the diseases. The form was closed at the time of the calf’s weaning or death and was collected by the technician during the monthly visit.

Body weight at birth and weaning was obtained indirectly with the use of chest tape (Chest tape for weighing cattle by https://site.multitecagro.com.br/pecuaria/, access 1 July 2024), with different scales for small (Jersey), medium (Holstein crosses with Jersey, Guernsey), and large (Holstein, Brown Swiss) breeds. Weight was read directly from the tape that contained the following scales: small animals 27–513 kg, medium 32–556 kg, and large animals 37–1005 kg. Heifers born below the scale measures were weighed with scales. This method is considered practical for farm studies where scales are unavailable or too expensive to purchase [21].

Morbidity was assessed by the symptoms of the main syndromes/diseases affecting the calves (diarrhoea, respiratory diseases, omphalopathy/sepsis, unspecified tick-borne parasitic disease/yellow sickness, or trauma) (Table 2). For this, each farmer received a chart with typical symptoms for each syndrome/disease to be evaluated.

### 2.3. Statistical Analysis

The dataset was visually inspected and manually cleaned to ensure all data entries were uniform and correct. When necessary to facilitate analysis, but without losing the biological meaning of the variables, the data were summarised, categorised, or re-categorised. The number of observations differed between analysis of the different response variables under investigation (perinatal mortality, postnatal mortality, morbidity, and weight gain). Observation differences were due to calf death or lack of information/data completeness. Once the final dataset was obtained, a decision tree of possible predictors associated with the responses was constructed to guide the analysis. The association of each predictor with the different response variables was evaluated in univariable multilevel models, where farm identification was used to indicate the interdependence of data from calves from the same farm. Thus, there were two levels present in the univariable models and subsequently in the multivariable modelling. The first level represents the calf and the second level represents the farm, added as a random term in the model.

Predictors associated with the response variable in the univariable analysis (*p* ≤ 0.20), were tested in multivariable models [19] when feasible. Potential explanatory variables were assessed for collinearity and not included in the further models when correlation was >0.6; in these cases, the variable with most biological relevance was kept. For the multivariable models, fixed effect variables were removed from the maximum multivariable model, prioritising the least significant ones, using manual backwards elimination of variables with *p*-value > 0.05. To control for confounding variables, after dropping any variable, changes in the coefficients of the remaining predictors were inspected. Changes in the coefficients greater than 30% were considered evidence of a confounder, and these variables were kept in the model. To accommodate for the different periods of calf risk due to different ages at weaning among farms, age at weaning centred on the mean was added to the models (except for perinatal mortality). Regardless of significance, age at weaning remained in the model until all others were significant (*p* < 0.05). At the end, if the age at weaning was significant, it remained in the model; otherwise, it was removed. Two-way interaction terms were tested among the variables in the final model using forward selection and dropped if their *p*-value > 0.05. Normality (Q-Q plots) and homoscedasticity of residuals from higher-level effects (fitted plots) were assessed graphically. For binary outcomes, overdispersion was checked using Pearson residuals. We also used the AIC (Akaike Information Criterion) to compare models. We used 12 points for adaptive quadrature estimation in order to improve the approximation of all the regressions; the *p*-values of all models were obtained by the Type II Wald Chi-Squared test. All analyses were performed with Software R (R Development Core Team 2017. A language and Environment for Statistical Computing), the *graphics*, *car*, and *lme4* packages. A total of 24 predictors were evaluated in the different analysis, as shown in Table 3, Table 4 and Table 5. Details of each analysis are found in the next paragraphs.

#### 2.3.1. Mortality

The association of the factors (predictors) with mortality (response variable) was tested with the multivariable mixed logistic regression model, *glmer* function of the *lme4* package [28], with farm as a random effect. Two types of mortality were evaluated, as described below:

##### Perinatal Mortality

Every calf born dead or dying within the first 24 h of life was considered a case of perinatal death. Due to the low number of death cases, a multivariable multilevel model would not have the power to identify all variables correctly. Therefore, only the first step of the analytical process was performed, that is, a univariable analysis with calf data grouped by farm. This analysis was performed to identify individual associations with this type of mortality.

The perinatal mortality rate was calculated from the total number of calves that died within 24 h in relation to the total number of calves born (dead + alive) during the study period.

##### Postnatal Mortality

Any calf death occurring between 25 h of life and weaning was considered a case of postnatal death. The postnatal mortality rate was calculated by dividing the total number of calves that died in this period by the total number of calves born alive in the 12 months of the study.

Initially, a mixed multivariable model was run, but as the final model did not show sufficient variance in all farms to justify the use of a mixed model with farm as a random term, a simple logistic regression model (univariable analysis) was used, and these results are presented here.

#### 2.3.2. Morbidity

Every calf affected at least once by any disease during the suckling period was considered a morbidity case. The association of potential factors (predictors) with morbidity (response variable) was tested with the mixed multivariable logistic regression model (*glmer*), with farm as a random effect. The morbidity rate was obtained by dividing the total number of calves that became ill at least once between the 25th h of life and weaning by the total number of calves alive at the beginning of the period.

#### 2.3.3. Weight Gain

Daily weight gain (g/d) was calculated from the difference between weaning weight (kg) and birth weight (kg), divided by age at weaning (d). The association of potential factors (explanatory variables) with weight gain (response variable) was tested with the linear mixed model, *lmer* functions from the lme4 package [28], with farm as a random effect. Calves that died and calves with incomplete data were excluded from the weight gain analysis.

## 3. Results

A total of 70/135 farms remained in the project until the end. Twenty-four farms dropped out due to labour issues, illness within the family, or the family giving up dairy farming. Another 41 were excluded from the study due to the low number of births of female dairy calves (<4), which was due to the following reasons: too many males were born, some cows did not become pregnant or aborted, and the farmers inseminated the cows with beef breeds (a common practice in the AUG region, which in the period of the study intensified due to the fall in milk prices and high meat prices). Animals in 67/70 farms were managed on pasture, using silage and concentrate as feed supplement in the trough. The three exceptions had the cows on free-stalls (n = 2) or compost barn (n = 1). Five farms had a closed maternity pen, while the rest of the farms (n = 65) had cows calving in open paddocks.

The farms had a median of 25 (range 9–70) lactating cows and a milk yield of 411 (range 96–1631) L/d. The participating families consisted of 4 (median, range 2–14) people, 45% women and 55% men. Most (57%) had incomplete elementary school, and only 3% had completed higher education. Farms with up to 50 ha prevailed (82.3%). On average, 39% of the farm area was used for dairy farming. The annual gross income of the farms was R$20,2871 (47,700–98,944, median and range), equating to USD 42,226, with 60% coming from the dairy activity. Of the total infrastructure investments made in the farms, 57% was related to milking and 5% to heifer rearing. The remaining investments were made in pig farming, poultry farming, agribusiness, fruit growing, and especially annual crops (soybean, corn, and wheat).

During the study period, 547 female calves were born, 538 (98%) alive and 9 (2%) dead. The median value of births was 6 per farm (4 to 37 range/farm). The mortality, morbidity risks, and weight gain (g/d) observed for each category of potential explanatory variables related to genetics, hygiene, and health are listed in Table 3; those related to nutrition are listed in Table 4; and those related to housing are listed in Table 5.

None of the participant farms pasteurised colostrum or had a colostrometer or refractometer, and only 11.4% stored surplus colostrum. In addition, none of the farms provided colostrum replacements to calves.

### 3.1. Mortality

**Figure 1:**
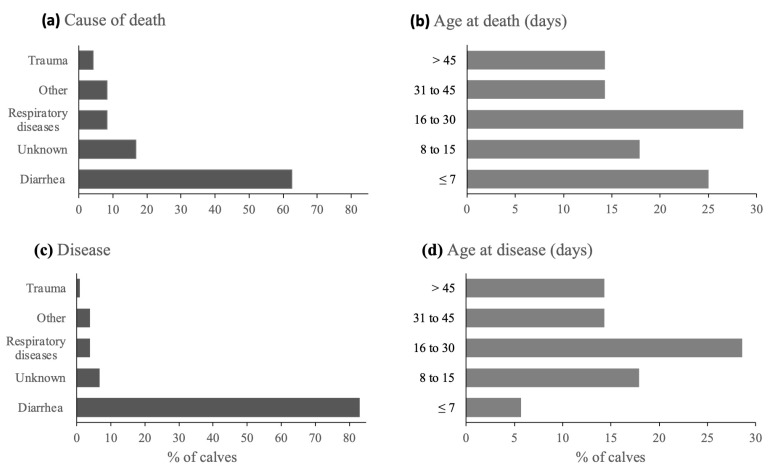
Cause (**a**) and age (**b**) of death (n = 538) and number of cases (**c**) and age (**d**) of disease (n = 534) in pre-weaning female dairy calves in 70 family farms in southern Brazil. Other = unspecified tick-born parasitic disease, intoxication, or hernia.

The total mortality risk was 6.8% (n = 37/547, median 0, range 0–50/farm), perinatal mortality risk was 2.4% (n = 13/547, median 0, range 0–50/farm) (≤24 h), and postnatal mortality risk was 4.4% (n = 24/534, median 0, range 0–40/farm). At the herd level, 30% (n = 21/70) of herds experienced at least one calf death during the study. In 73% of farms, no postnatal death occurred, while in 11%, mortality was 20%. Diarrhoea was the most reported cause of death, followed by unknown causes and respiratory diseases (Figure 1a). Most deaths occurred in the first month of life (Figure 1b).

#### Individual Factors Associated with Perinatal and Postnatal Mortality

Calves born from dystocia were more likely (OR = 5 CI 1.1–21.3; *p* = 0.03) to be born dead or to die within the first 24 h of life. Calves that had at least one case of diarrhoea between the second day of life and weaning were more likely (OR = 10.02 CI 4.08–24.65; *p* < 0.001) to die than calves that never got sick. Additionally, the probabilities of dying (OR = 0.77, CI 0.67–0.89; *p* < 0.001) decreased each day with increasing age at weaning.

### 3.2. Morbidity

Of the 534 calves alive after 24 h of life, 106 (19.9%) fell ill at least once until weaning. Diarrhoea was the disease with the largest number of cases, followed by unknown causes (Figure 1c). Most cases of disease occurred up to 30 days of age (Figure 1d).

#### 3.2.1. Factors Associated with Postnatal Morbidity

Birth season and weaning age were significantly associated with pre-weaned heifer calf morbidity, with an interaction between the two factors. The probability of a pre-weaned heifer calves becoming ill decreased by 10% with each additional day of life and was influenced by season of birth (Table 6). Calves born in the cold season (winter/spring) were almost six times more likely to fall ill than calves born during the warm season (summer/autumn).

#### 3.2.2. Weight Gain

Calves (n = 505) were born at an average birth weight of 40 ± 8 kg and were weaned at 69 ± 16 days, weighing 80 ± 20 kg, which corresponds to a gain of 570 ± 212 g/d (mean and SD). The weight gain of Holstein, Jersey, and other breeds of calves was, respectively, 605 ± 208, 471 ± 206, and 452 ± 198 g/d.

#### 3.2.3. Factors Associated with Weight Gain

Jersey calves and other breeds gained less weight than Holstein calves. Calves from herds that used milk replacer had a lower weight gain than calves from herds that used milk. On the other hand, heifers from herds receiving more than 4 L/d of milk and 0.5 kg/d or more feed had greater weight gain than heifers from herds receiving smaller amounts of milk (Table 7).

#### 3.2.4. Variance Partition of Multivariate Models

Table 8 shows the variance partition for calf morbidity and weight gain in the 70 farms. In the final models, the factors that most influenced morbidity were in farm-level variables, while for weight gain, they were in calf-level variables.

## 4. Discussion

This is the first known prospective study assessing mortality, morbidity, and weight gain and associated factors in pre-weaning female dairy calves in smallholder grazing farms in Brazil. The models indicate that the farm-level variability for both morbidity and weight gain was significant. In addition, they indicate that morbidity is more dependent on the farm than calf characteristics and that, on the other hand, weight gain is more dependent on the characteristics of the calf than the characteristics observed in the herd/farm.

### 4.1. Mortality

The mortality rates observed in this study can be considered relatively low, considering international references (perinatal: 2–20% [29]; postnatal: 5–11% [30]), and similar to a study conducted in the same region [15]. This may be related to the low number of calves raised by farm in the period (on average 7.7), which may have facilitated the care and individual observation of the animals. For example, the presence of more calves on a farm increases the possibility of calves being housed in groups, which in turn increases the chance of transmission of contagious diseases, which are more common the larger the group of animals [31,32].

In 73% of farms, no postnatal death occurred, while in 11%, mortality was 20%. These results corroborate other studies showing similar variability [15,30,32], indicating that average mortality values obscure the fact that herd-level statistics follow an asymmetric distribution to the right, as most herds have no or minimal losses, while some herds have mortality above 20% [33]. This disparity underscores the diversity in management practices, highlighting the potential for enhancement in the farms across the region.

The significant association between dystocia and perinatal mortality identified in the univariate model reinforces the need for qualified calving assistance and attention in choosing the bull, especially considering the ease of calving. Reports from the literature indicate that dystocia is a major cause of perinatal mortality [34]. Dystocia, or difficult birth, can lead to complications such as trauma, hypoxia, and prolonged labour, which weaken calves and increase their susceptibility to infections. Similarly, inadequate colostrum management is critical, as it affects passive immunity transfer, leaving calves more vulnerable to diseases early in life. In this study, mortality up to the first week of life was 3%, and combined with 14% of dystocia events, it indicates that these factors play a significant role in calf survival.

The occurrence of the highest number of calf deaths in the first weeks of life suggests a failure in the transfer of passive immunity via colostrum. Colostrum management is the factor that most influences the health and survival of calves, given that calves under five weeks of age have limited adaptive immunity and colostrum immunoglobulins, such as IgG, are their primary source of antibodies to protect them from infectious diseases during this phase of life [35,36]. While calves can mount an early innate immune response, their full adaptive immune system develops gradually. Inadequate colostrum intake or quality are main factors of increased morbidity and mortality [37,38]. Therefore, ensuring the ingestion of appropriate volumes of high-quality colostrum within the first few hours after birth is a crucial calf management tool [35]. Additionally, feeding colostrum in high concentrations for a short period or in lower concentrations for an extended period beyond the first day of life has been shown to positively affect weight gain, reduce the risk of diarrhoea, and lower mortality [39]. None of the participants in this study had any immune, nutritional, or sanitary control of colostrum. The probability of passive immunity transfer failure is higher when there is no routine monitoring on the farm [40], as the concentration of IgG in colostrum is extremely variable (<1 to 235 g/L; [41,42,43]). Therefore, IgG concentration should be estimated regularly in calves after the first 24 h of life to test compliance with colostrum management. Colostrum IgG content can be easily determined on the farm from the total whey protein using a refractometer [44]. In this study, not only did participants fail to evaluate the quality of colostrum but only 11.4% stored colostrum, and little care was given to the amount offered. In addition, farmers seemed unaware of the implications of delaying the neonate’s first ingestion of colostrum (see the companion study [16]).

Regarding the relationships between management factors and mortality, it is possible that the final sample size might have been insufficient to identify significant differences among the assessed predictor variables in the multivariable model. For reasons explained earlier, 48% of the farms that started the study were not included in the analysis, which reduced the power of the sample. In addition, mortality has multiple causes, which makes it difficult to identify specific factors, especially in observational studies involving farms where there is no systematic and controlled rearing system, as in the present study. Other research, even with a much larger number of calves, has failed to identify the management factors that have greatest influence on animal mortality [30].

### 4.2. Morbidity

Diarrhoea was the main disease affecting calves, confirming reports of the literature [1,3,6,45,46], including in Brazilian farms [13,14,15]. Important factors related to diarrhoea are colostrum, milk intake, housing type, housing hygiene, and infectious diseases [44,47,48,49]. The low volume of colostrum provided, coupled with the large number of cases of diarrhoea observed in the present study, suggest that in many farms, the amount and quality of colostrum supplied to calves was not adequate.

Milk quality and quantity may also have influenced the number of cases of diarrhoea. In addition to the fact that 74% of participant farms provided a maximum of 4 L/d of milk and, while most used waste milk (e.g., milk from cows with mastitis or from the transition period) to feed the calves, none of them pasteurised the milk. The high bacterial load of waste milk has been associated with an increase in diarrhoea in calves. Thus, pasteurisation may be beneficial for maintaining animal health [50] and weight gain and may result in lower morbidity and mortality rates [51]. In addition, milk from cows with mastitis usually contains high concentrations of antibiotics, which can affect heifer health, generate resistant bacteria, and contaminate the environment through the presence of drug residues in faeces and urine [52]. The average amount of milk provided, equivalent to 10% of birth weight and 5% of weaning weight, is well below the daily needs of calves [53,54,55]. Previous studies have found that the daily amount of milk consumed influences the solid feed intake, growth rate, and health of calves [49,56,57].

The greater morbidity during winter may be related to the most adverse weather conditions at this time of the year in southern Brazil. The incidence of disease increases in calves subjected to a cold climate [47]. Normally, in the winter months (June to August in southern Brazil), there are days of intense cold with minimum temperatures of 10 °C and reaching −3 °C, accompanied by heavy and persistent rainfall with monthly averages of 150 to 250 mm. The interaction of weaning age with season detected in this study is possibly due to the fact that heifers go through a period of low immunity around the fourth week of life [36]. At this age, the passive immunity acquired with colostrum is low, while the immunity developed by the heifer is still incipient. Therefore, it is possible that the most stressful conditions of winter (lower temperatures, higher humidity, and wind) increased the number of disease cases in this period of the calves’ life. If that is true, four- to six-week-old calves would fall ill more in winter than in summer, maintaining a more persistent morbidity rate during the cold period of the year.

### 4.3. Calf Weight Gain

Weight gain in the present study was lower than in other studies, which reported growth rates (in g/d) of 596 [6], 615 [58], 790 [7], and 950 [11]. Possible reasons for the lower weight gains found in the present study are diarrhoea, malnutrition, and poor colostrum management practices (e.g., timing of colostrum administration) [6,7,59]. In 44% of the farms, calves did not ingest any colostrum before 2 h of life, and those who ingested the first colostrum within 2 h after birth gained 85 g/day more than those who ingested colostrum only after 2 h of life. The ingestion of colostrum with a higher IgG concentration has been associated with higher weight gain in dairy animals [60,61].

The participating farms followed management practices commonly found in dairy farms [62,63], including in the region [13,14], such as separation from their mothers soon after birth and restricted amounts of milk, or replacer provided in a bottle or bucket. As expected, the daily amount of milk and feed provided were positively related to heifer weight gain. In almost half of the farms studied, calves received less than 0.5 kg/d of feed, possibly insufficient to supplement the daily nutrient requirements for maintenance and weight gain obtained from milk or milk replacer. Calves that suckle or are fed ad libitum consume approximately 20% of body weight in milk each day, resulting in daily weight gains of up to 1 kg. In addition to better feed efficiency, calves that receive more milk also tend to show improved welfare [64,65]. By contrast, low feed intake can weaken the immune system and reduce weight gain [65,66].

The quality of milk replacers was not evaluated in this study. However, the fact that calves fed replacement gained less weight suggests poor product quality, poor preparation or delivery, or a lack of adaptation of the calves. Lower-quality or poorly prepared substitutes, supplied in large quantities without calf adaptation, can cause diarrhoea and consequently affect animal development [67].

In dairy production systems, heifers are typically responsible for approximately 20% of the costs and are the second largest expense after feeding lactating cows [68]. It has been shown that farmers in the region who work with pure breeds were generally more skilled and invested more time and money in heifer rearing [16]. However, low investments in the rearing system may have negatively affected calf performance. Finally, the issues raised in this paper are critical for young dairy cattle welfare [69], which has been shown to be a public concern [70,71]. Farmers in the region have been shown to give low relevance to the welfare of dairy calves [14,16]. A change in this perception is needed, as not considering the public’s values may erode the industry’s social license.

The power of the results presented here and the depth of the analysis are limited by the number of participating farms and the ability to collect detailed data consistently across time. The reality of the study region, with a high density and heterogeneity of family farmers with low technification, makes it difficult to characterise and accurately diagnose management problems to tackle. When achieved, it requires balancing time, labour, and citizen participation, with the respective caveats. A concern about this balancing act is the classification of diseases. We opted for using practical and clear definitions to minimise misinterpretation, considering the region’s operational challenges. While this approach aimed to ensure consistency, we acknowledge that some misclassification is possible, particularly where overlapping symptoms could indicate multiple conditions, such as lethargy. However, these definitions were based on the established literature and reflect the realities of field data collection. There has been a paucity of knowledge about calves’ welfare in the region, and this is one of the first longitudinal reports on mortality and morbidity. Despite this study limitations (sample size and reliance on potentially imprecise recording from farmers), we have highlighted important areas to consider in the future by local producers and areas where research is needed. Furthermore, we calculated the study’s power based on the observed total mortality risk of 6.8% and a 2% effect size. With 547 calves across 70 farms and an intra-class correlation (ICC) of 0.1, we achieved approximately 84% power, which is above the conventional 80% threshold. This supports our study capacity to detect meaningful effects within the study’s design despite the limitations found.

## 5. Conclusions

The calf morbidity and mortality rates found in this study are similar to those of other international studies, despite the many potential differences in size, infrastructure, environment, and management of farms and the herd. In summary, the results of the present study suggest that the quality of heifer management in many of the surveyed farms may be compromising the health, performance, and survival of heifers. This has implications for animal welfare and possibly the productivity of dairy herds in the state. Weight gain was below that reported in the literature. The findings suggest that colostrum and milk feeding, as well as the quality of the rearing environment are major challenges to be overcome in the studied region and may help explain the relatively poor productive performance and economic viability of dairy activity in many farms in the state. They can be useful for future comparisons between farms, as well as to support extensionists, consultants, and dairy farmers and to guide official programs.

## Figures and Tables

**Table 1:** Criteria adopted to classify the hygiene, humidity, insolation, and ventilation of calf housing.

Item	Good	Average	Poor
Hygiene	Clean environment, without the presence of faeces, mud, waste, and flies	Presence of some mud, faeces, or waste, covering a maximum of 50% of the floor	Very dirty, presence of mud, faeces, or dirt on more than 50% of the floor
Humidity	Dry place	Wet place, water-saturated soil, sticky surfaces	Accumulation of water or mud on the surface
Insolation	The animal chooses when to stay in the sun, north–south ridge	Shaded, low, small, or no windows, east–west ridge	No access to solar radiation
Ventilation	Huts, if stall, ≥6 m^3^ air/heifer, two sides open, no unpleasant odour	Weak air currents, temperature above the outside, slight unpleasant odours	Closed, stuffy environment with strong, unpleasant odours

**Table 2:** Typical symptomatology of syndromes/diseases monitored by farmers.

Syndrome/ Disease	Symptoms	Source
Diarrhoea	Watery stools, inappetence, cold extremities, lethargy/apathy, prostration/weakness, and dehydration	[22,23]
Respiratory Diseases	Eye discharge, nasal discharge, drooping ears, crooked head, spontaneous cough, rapid or difficult breathing, and rectal temperature above 39.2 °C. Total score ≥ 5 was considered a case of bovine respiratory disease (Supplementary Materials)	[24,25]
Omphalopathy/ Sepsis	Swollen, painful, pus-filled umbilicus, swollen joints, muscle pain, and petechiae (spots) on mucous membranes	[26]
Unspecified tick-born parasitic disease/Yellow sickness	Anaemia, jaundice, and weight loss	[27]

**Table 3:** Postnatal mortality, morbidity, and weight gain of pre-weaned heifer calves, observed in herds in southern Brazil, are distributed by the categories of potential predictors: genetics, hygiene, and health.

Variable	Analysis Inclusion ^1^	Category	Postnatal Mortality ^2^ n = 534		Morbidity n = 534		Weight Gain n = 505	
			n	%	n	%	n	g/d
Dam’s parity	a, b, c, d	1	107	4.7	107	20.6	101	580
		2	99	5.1	99	21.2	94	581
		≥3	328	4.3	328	19.2	310	563
Breed of sire	a, b, c, d	Holstein	405	4.4	405	18.3	382	605
		Jersey	66	6.1	66	31.8	62	471
		Other	63	3.2	63	17.5	61	452
Calving	a, b, c, d	Dystocia	69	5.8	69	26.1	65	525
		Normal	465	4.3	465	18.9	440	576
Hygiene of the delivery site	a, b, c, d	Good	132	3.0	132	23.5	127	576
		Bad	402	5.0	402	18.7	378	568
Time spent with the dam	a, b, c, d	≤12 h	76	3.9	76	21.1	72	504
		>12 h	419	4.5	419	20.3	396	586
		Unknown	39	5.1	39	12.8	37	525
Healing of the navel	b, c, d	On	173	3.5	173	22.0	166	498
		Yes	361	5.0	361	18.8	339	605
Diarrhoea (at least one case)	b, d	On	446	2.0	*	*	434	577
		Yes	88	17.0	*	*	71	524
Birth season	a, b, c, d	WS ^3^	286	3.5	286	25.5	273	563
		SA ^4^	248	5.6	248	13.3	232	578

^1^ Univariable model in which the variable was tested: a = perinatal mortality; b = postnatal mortality, c = morbidity, d = weight gain; ^2^ 25 h weaning; ^3^ WS: winter/spring; ^4^ SA: summer/autumn; * not tested.

**Table 4:** Postnatal mortality, morbidity, and weight gain of dairy calves observed in herds in southern Brazil are distributed by category of potential predictors related to nutrition.

Variable	Analysis Inclusion ^1^	Category	Postnatal Mortality ^2^ n = 534		Morbidity n = 534		Weight Gain n = 505	
			n	%	n	%	n	g/d
Time to first intake of colostrum	a, b, c, d	≤2 h	306	4.6	306	20.9	288	607
		>2 h	228	4.4	228	18.4	217	521
Amount of ingested colostrum up to 24 h of life	b, c, d	≤4 L	239	5.4	239	20.9	225	571
		>4 L	196	4.1	196	19.4	188	568
		Unknown	99	3.0	99	18.2	92	571
Form of administration of colostrum	b, c, d	Artificial	401	4.0	401	19.0	381	575
		Mixed	73	5.5	73	19.2	68	588
		Natural	60	6.7	60	26.7	56	514
Type of milk	b, c, d	Milk	484	4.3	484	19.6	458	577
		Milk replacer	50	6.0	50	22.0	47	497
Form of administration of milk	b, c, d	Other	148	4.1	148	20.9	137	568
		Teat	386	4.7	386	19.4	368	570
Quantity of milk	b, c, d	≤4 L/d	371	4.9	371	19.4	349	530
		>4 L/d	163	3.7	163	20.9	156	660
Amount of feed (concentrate)	b, c, d	<0.5 kg/d	210	4.3	210	23.8	201	497
		≥0.5 kg/d	324	4.6	324	17.3	304	618

^1^ Univariable model in which the variable was tested: a = perinatal mortality; b = postnatal mortality, c = morbidity, d = weight gain; ^2^ 25 h weaning.

**Table 5:** Postnatal mortality, morbidity, and weight gain of dairy calves observed in herds in southern Brazil are distributed by category of potential predictors related to housing.

Variable	Analysis Inclusion ^1^	Category	Postnatal Mortality ^2^ n = 534		Morbidity n = 534		WG n = 505	
			n	%	n	%	n	g/d
Housing system	b, c, d	Collective	188	5.9	188	23.4	176	557
		Individual	346	3.8	346	17.9	329	577
Type of accommodation	b, c, d	Bay	334	5.1	334	19.2	312	580
		Another	200	3.5	200	21.0	193	553
Disinfection of the environment	a, b, c, d	No	475	4.8	475	19.2	450	563
		Yes	59	1.7	59	25.4	55	629
Bed	b, c, d	No	426	4.5	426	21.1	405	557
		Yes	108	4.6	108	14.8	100	620
Hygiene	b, c, d	Good	106	2.8	106	19.8	99	666
		Average	311	5.1	311	16.1	294	564
		Bad	117	4.3	117	29.9	112	499
Insolation	b, c, d	Good	172	2.9	172	18.6	166	596
		Average	161	5.6	161	22.4	152	572
		Bad	201	5.0	201	18.9	187	545
Floor	b, c, d	Another	400	5.5	400	22.2	374	549
		Ripped	134	1.5	134	12.7	131	631
Humidity	b, c, d	Good	318	3.1	318	16.7	303	580
		Bad	216	6.5	216	24.5	202	555
Ventilation	b, c, d	Good	372	3.8	372	16.7	357	571
		Bad	162	6.2	162	27.2	148	568

^1^ Univariable model in which the variable was tested: a = perinatal mortality, b = postnatal mortality, c = morbidity, d = WG; ^2^ 25 h weaning.

**Table 6:** Mixed multivariate generalised linear model, with farm as a random effect, for factors associated with postnatal morbidity (25 h—weaning) of pre-weaned female calves in family farms in southern Brazil (n = 534 heifers, 70 farms).

Variable	Category	OR	OR 95% CI	*p*-Value	
				Category	Variable
Intercept		0.04	0.02–0.09		<0.001
Season of birth	S/A ^1^	Reference			0.001
	W/S ^2^	5.83	2.71–12.55	<0.001	
Age at weaning		0.90	0.86–0.94	<0.001	<0.001
Season of birth: Age at weaning ^3^		1.08	1.03–1.13	0.001	0.001

^1^ S/A: summer/autumn. ^2^ W/S: winter/spring. ^3^ Age at weaning centred on the average.

**Table 7:** Multivariable linear mixed model, farm as a random effect, for the variables with significant association with weight gain (g/d) of calves in farms in southern Brazil (n = 505 calves ^1^, 70 farms; Coef = coefficient).

Variable	Category	Coef	95% CI	*p*-Value	
				Category	Variable
Intercept		519	464–573		<0.001
Breed of sire	Dutch	Reference			<0.001
	Jersey	−86	−140–−32	0.002	
	Another	−118	−180–−56	<0.001	
Liquid diet	Milk	Reference			0.02
	Milk replacer	−82	−149–−15	0.02	
Amount of liquid diet	≤4 L/d	Reference			0.002
	>4 L/d	115	41–189	0.003	
Amount of feed	<0.5 kg/d	Reference			0.02
	≥0.5 kg/d	79	13–144	0.02	

^1^ All calves that died (n = 37) and with incomplete data (n = 5) were excluded.

**Table 8:** Variance partition, mixed generalised linear model for morbidity, and mixed linear model for weight gain, family farms as a random effect, for dairy herds in 70 farms of southern Brazil.

Model	Source of Variation	Morbidity	Weight Gain
Null	Calf	53.2	53.1
	Farm	46.8	46.9
Final	Calf	46.0	61.4
	Farm	54.0	38.6

## Data Availability

The datasets generated for this study can be made available, upon request to the authors.

## Supplementary Materials

The following supporting information can be downloaded at https://www.mdpi.com/article/10.3390/ani14223327/s1; the recording sheet is available to farmers for heifer follow-up. Reference [72] are cited in the supplementary materials.
