# Supplementary material for: Farm and Animal Factors Associated with Morbidity, Mortality, and Growth of Pre-Weaned Heifer Dairy Calves in Southern Brazil
Source: Animals (Basel). 2024 Nov 19;14(22):3327. doi: 10.3390/ani14223327 (PMC11591025; doi:10.3390/ani14223327)
Supplement: Supplementary file 1 [file animals-14-03327-s001.zip › animals-3216677-supplementary.pdf]

Recording sheet available to farmers for heifers follow up

Sheet 1. Female calf growth monitoring sheet.

**Name of the farmer:**			Municipality:			²number:
**HEIFER SHEET**						
**Ear tag:**	Twins ☐	Mother:		Father:	Breed:	
**Mother's data:**	Vaccinated	☐ Yes	☐ No	How many calvings?		¹BCS
**Calving:**	☐ Normal	☐ Mild problems Assistance of 1 person		☐ Serious problems Assistance of 2 or + people	☐ Mechanical or surgical intervention	
**Birth**	☐ Normal		☐ Dead		☐ Died within 24 h	
**Colostrum:**	☐ Colostrum	☐ Replacer	☐ Dam	☐ Nipple	☐ Bucket	☐ Mixed
**Aleitamento:**	☐ Milk	☐ Replacer	☐ Dam	☐ Nipple	☐ Bucket	☐ Mixed
**Habitação:**	☐ Individual	☐ Group	☐ Hut	☐ Indoor Pen	☐ Outdoor Pen	☐ Rope
**Nascimento:**	Date __ / __ / ___		Weight		Height	
**30 days of life**	Date __ / __ / ___		Weight		Height	
**Weaning:**	Date __ / __ / ___		Weight		Height	¹ECC
**In case of death:**	Date __ / __ / ___		Weight		Height	

¹BCS: Body condition score (1 = Very thin, 2 = Very fat), ² ¹BCS: Body condition score (1 = Very thin, 2 = Very fat), ² Farm number

Sheet 2. Female calf health monitoring sheet Part 2

	**DATE**						
	**DISEASE**						
**MEDICINES**	Name						
	Doses (ml or g)						
	Number of doses						
	Value (R$/bottle)						
	Name						
	Dose (ml or g)						
	Number of doses						
	Value (R$/bottle)						
**SYMPTOMS**	Liquid faeces						
	Does not eat						
	Cold extremities						
	Lethargy/Apathy						
	Prostration/weakness						
	Dehydration (Deep eye)						
	Eye discharge						
	Nasal discharge						
	Droopy ears/Crooked head						
	Spontaneous cough						
	Fast/Hard breathing						
	Temperature > 39.2ºC						
	Swollen navel/Pain						
	Navel with pus						
	Swollen joints						
	Lameness						
	Spots on the mucous membranes						
	Anemia						
	Jaundice						
	Weight loss						

Sheet 3. Scoring system for respiratory symptoms of pre-weaned dairy calves. [2] Any abnormality, not limited to the examples above. If the total score of the heifer is ≥ 5, it may be a BRD case: LOVE et al, 2014; ALY et al., 2014.

Normal score / No clinical sign		Clinical sign / Abnormal score (any severity)[2]					
0 if No Eye discharge / No abnormalities	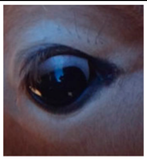	Score of 2 if	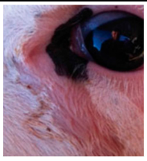	or	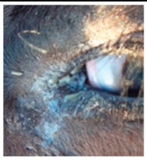	or	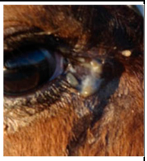
0 if there is no nasal discharge	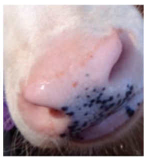	Score of 2 if	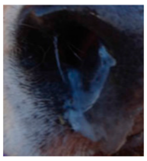	or	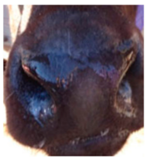	or	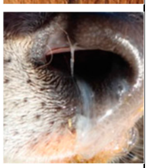
0 if No Droopy ears or tilted head	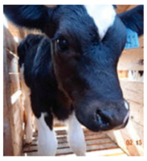	Score of 2 if	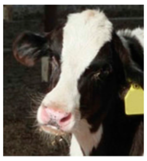	or	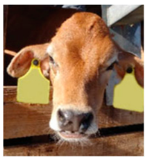	or	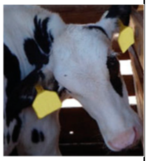
0 if No Cough		Score of 2 if there is any spontaneous cough					
0 if Normal Breathing		Score of 2 if breathing is quick or difficult					
0 if Normal Temperature		Score of 2 if rectal temperature is ≥ 39.2 ºC					

Sheet 4. (<u>Front page</u>/Recording sheet). Sepsis diagnosis scoring system. Source: (Fecteau et al., 1997).

Evaluation criteria		Observed	Points
A. The site is a source of infection[1]		No	0,0
		Yes	1,5
B. Age of the heifer		< 7 days	0,0
		≥ 7 days	1,2
C. Clinical score	Moisturisation	0 1 2 3	
	Sclera (white of the eye)	0 1 2 3	
	Attitude	0 1 2 3	
	Navel	0 1 2 3	
	Faecal	0 1 2 3	
	Total clinical score		
C. Clinical score class		< 5	0,0
		5 - 8	2,1
		> 8	2,5
CLINICAL SEPSIS SCORE (A+B+C)			

[1]Presence of hypopyon, septic arthritis, soft tissue abscess, or mucopurulent nasal discharge indicates that the site is a focus of infection.

Sheet 4. (<u>Back page</u> /Description sheet) - Sepsis diagnosis scoring system. Source: (Fecteau et al., 1997).

Type of observation	Symptoms to be observed and related score
Level of Hydration	Eyeball position, nasal mucosa, skin turgor test
0	Normal hydration, skin turgor less than 2 s.
1	Moderate dehydration, slightly sunken eyeball and skin turgor 2 to 4 s.
2	Apparent dehydration, sunken eyes, dry nose, skin turgor ≥ 5 s.
3	Severe dehydration, very deep-set eyes, with an easily perceptible distance between the eyeball and the eyelids, persistent skin turgor
Scleral vessels	Number, size, colour and proximity to the limbus of the vessels of the sclera
0	Maximum 1 pot, it does not reach limbus
1	2 to 4 pots, at least 1 reaches the limbus; colour is still pink, size is normal
2	5 to 6 vessels, at least 2 reach the limbus, colour is red, size is slightly increased.
3	> 6 vessels, at least 3 reach the limbus, colour is purple, size is significantly increased.
Heifer attitude	Behaviour, skills and attitude
0	Normal behaviour, alert, stands up when someone approaches, interested in surroundings.
1	Depressed, must be stimulated to get up.
2	He gets up only with help.
3	Unable to stand, even with help.
Navel	Pain, size, dryness, presence of pus
0	Normal, pencil size, dry and painless.
1	Larger than normal, but dry and painless
2	Bigger than normal, wet or painful.
3	Larger than normal, with purulent discharge and evidence of pain (any presence of internal umbilical swelling should be scored as a 3)
Faeces	Consistency, blood presence
0	Normal faeces
1	Softer stools than normal but no diarrhoea in the tail
2	Diarrhoea, but not profuse, wet tail
3	Profuse watery diarrhoea, wet tail, dirty stall, or any evidence of blood or fibrin in the faeces (but not normal faeces with a small amount of blood).

Fecteau, G., Pare, J., Van Metre, D.C., Smith, B.P., Holmberg, C.A., Guterbock, W., Jang, S., 1997. Use of a clinical sepsis score for predicting bacteremia in neonatal dairy calves on a calf rearing farm. The Canadian Veterinary Journal 38, 101.
